# Exploration and validation of the influence of angiogenesis-related factors in aortic valve calcification

**DOI:** 10.3389/fcvm.2023.1061077

**Published:** 2023-02-07

**Authors:** XiangJin Kong, LingWei Meng, KaiMing Wei, Xin Lv, ChuanZhen Liu, FuShun Lin, XingHua Gu

**Affiliations:** ^1^Qilu Hospital, Cheeloo College of Medicine, Shandong University, Jinan, Shandong, China; ^2^Department of Cardiovascular Surgery, Qilu Hospital of Shandong University, Jinan, Shandong, China

**Keywords:** calcific aortic valve disease (CAVD), angiogenesis-related factors, secretogranin II (SCG2), zinc finger E-box binding homeobox 1 (ZEB1), miRNA, transcription factors (TFs)

## Abstract

Over the years, bioinformatics tools have been used to identify functional genes. In the present study, bioinformatics analyses were conducted to explore the underlying molecular mechanisms of angiogenic factors in calcific aortic valve disease (CAVD). The raw gene expression profiles were from datasets GSE153555, GSE83453, and GSE51472, and the angiogenesis-related gene set was from the Gene Set Enrichment Analysis database (GSEA). In this study, R was used to screen for differentially expressed genes (DEGs) and co-expressed genes. Gene Ontology (GO) and Kyoto Encyclopedia of Genes and Genome (KEGG) Pathway enrichment analysis were performed on DEGs and validated in clinical samples. DEGs in CAVD were significantly enriched in numerous immune response pathways, inflammatory response pathways and angiogenesis-related pathways. Nine highly expressed angiogenesis-related genes were identified, of which secretogranin II (SCG2) was the most critical gene. MiRNA and transcription factors (TFs) networks were established centered on five DEGs, and zinc finger E-box binding homeobox 1 (ZEB1) was the most important transcription factor, verified by PCR, immunohistochemical staining and western blotting experiments. Overall, this study identified key genes and TFs that may be involved in the pathogenesis of CAVD and may have promising applications in the treatment of CAVD.

## 1. Introduction

Calcific aortic valve disease (CAVD) is a localized inflammatory infiltrative disease characterized by aortic valve calcium deposition and valve tissue fibrosis, may cause aortic stenosis, which may cause heart failure (1). Frequently, severe CAVD is linked to significant rates of mortality and disability (2). Although the cause of CAVD is uncertain, it has been determined that active aortic valve inflammation and lipid deposition play a significant role in the progression of the disease (3). Surgery and transcatheter aortic valve replacement remain mainstays of treatment for CAVD (4), highlighting a lack of therapeutic approaches that can slow disease progression (5).

There are several similarities between vascular calcification and atherosclerosis, including chronic inflammation and extracellular matrix (ECM) remodeling, angiogenesis, valvular interstitial cells (VICs) proliferation and differentiation, as well as calcific lesions development (6, 7). In addition to the aforementioned factors, the interaction of angiogenesis-related factors with active inflammation may play a significant role in the pathogenesis of CAVD; however, it is not known which angiogenesis-related genes play an impact in calcified valve tissue (8). Interestingly, although the heart is a blood-rich organ, there are no blood vessels in the normal heart valves (9). In mice, the progression of aortic valve calcification is slowed by the deletion of specific pro-angiogenic genes (10). Although studies in recent years have presented different perspectives on the similarities and differences between vascular calcification and atherosclerosis, especially in terms of neoangiogenesis, the consensus is that vascular calcification and atherosclerosis have more similarities than differences (11), but what cannot be ignored is that the factors related to angiogenesis, inflammation, lipid deposition and other related factors cross talk with each other, which may play an important role in the progression of aortic valve disease (7, 12). However, our current understanding of the relationship between CAVD and angiogenesis-related genes is still limited. Exploring this process may provide new insights into the treatment of CAVD.

In recent years, microarray technology combined with comprehensive assays have been used to identify novel functional genes associated with a variety of diseases (13, 14). These genes can be used in the diagnosis of various diseases and in the study of disease-related influences. Many teams in the CAVD field have used innovative bioinformatics research techniques to examine the disease’s potential affecting elements from various perspectives (15–17), but none of them explored from the specific perspective of angiogenesis-related factors in CAVD. In this study, we obtained three public microarray datasets (GSE153555, GSE83453, and GSE51472) and the Msigdb database of angiogenesis-related genes to explore meaningful pathogenic genes.

## 2. Materials and methods

### 2.1. Microarray data

The gene expression profiles for the datasets GSE83453, GSE51472, and GSE153555 were retrieved from the Gene Expression Omnibus (GEO) database. From samples of human tissue, each gene expression profile was created. An analysis of dataset GSE83453 was performed based on platform GPL10558 (Illumina HumanHT-12 V4.0 expression BeadChip), which includes nine calcified aortic valve samples and eight normal aortic valve samples. Dataset GSE51472 was based on the GPL570 (Affymetrix Human Genome U133 Plus 2.0 Array) platform and included 10 calcified aortic valve (there of 5 sclerotic valves with micro-calcification and 5 severely calcified valves) samples and 5 normal aortic valve samples. GSE153555, based on the GPL16791 platform, comprised 5 calcified and 5 normal aortic valve samples (measured in duplicates, thus *n* = 10 data points were depicted for calcified and normal valve samples, respectively). We screened genes closely related to angiogenesis from the Gene Set Enrichment Analysis (GSEA) database.^1^

### 2.2. Data pre-processing and DEGs screening

We used the “linear models for microarray data (limma)” package in R 4.0.1 to perform log2-transformation, background removal, and quantile normalization on the expression profiles of GSE83453, GSE51472, and GSE153555 (R Foundation for Statistical Computing, Vienna, Austria). According to the annotation file, probe IDs were converted into gene symbols. An average expression value was used for multiple probes mapping to a single gene. The batch effect was corrected using the “SVA” package’s combat feature, a widely used empirical Bayes method for batch correction. An adjusted *P*-value for multiple testing was applied to control false discovery rates. After pre-processing, the “limma” package screened DEGs between calcified and normal aortic valve samples with a threshold of adjusted *P*-value < 0.05 and | log2 fold-change (FC)| ≥ 0.5. Additionally, the volcano plots and heatmaps used to display the DEGs were created using “ggplot2” and “Pheatmap.”

### 2.3. Screening of DEGs and angiogenesis-related genes

Genes from the three datasets were intersected to identify the hub genes. 48 angiogenesis-related genes were downloaded from the GSEA database. Then, the angiogenesis-related genes were screened by intersecting with angiogenesis-related genes.

### 2.4. Functional analysis of DEGs

Gene Ontology (GO) and Kyoto Encyclopedia of Genes and Genomes (KEGG) pathway enrichment analysis was carried out using the “clusterProfiler” package in R to better understand the biological significance of DEGs.

### 2.5. The expression of hub genes in calcified or normal aortic valve sample

We used the online mapping site sangerbox in R to plot box plots of relative expression of genes for the three datasets. Wilcoxon rank-sum test was used to evaluate the expression of intersected genes in calcified and uncalcified aortic valves from GSE83453, GSE153555, and GSE51472. The box plots of hub gene expression were made using the ggplot2 and ggsignif programs.

### 2.6. Construction of the miRNA-target, TF-gene, and TF-miRNA gene network

To further explore putative miRNAs that target the intersected genes, we used the TarBase v8 database^2^ to establish a gene-miRNA interaction network, and a score ≥ 0.95 was considered as the cutoff criterion for predictive analysis. We only selected target mRNAs identified in the aforementioned databases for further analysis. To explore the relationship network between miRNA and transcription factors (TFs) of the screened key genes, we used the JASPAR^3^ and ENCODE^4^ databases for analysis and established an interaction network diagram. The above network was visualized by the online tool NetworkAnalyst.^5^

### 2.7. Establishment of the protein-chemical network of the key gene

To explore the relationship network between key genes and chemicals, we analyzed data from the Comparative Toxicogenomics Database (CTD)^6^ and established an interaction network diagram to explore the chemical complexes key genes can act on to provide new insights for future drug prediction.

### 2.8. Human research and ethics

Human calcified aortic valve (CAV) leaflets (A total of 10 distinct people’s samples were collected) were obtained from patients with CAVD undergoing aortic valve replacement. Normal aortic valves (A total of 10 distinct people’s samples were collected) in the control group samples from organ donations. Valves with congenital bileaflet aortic valves (BAVs), moderate to severe aortic regurgitation, infective endocarditis, congenital valve disease, and rheumatic aortic valve disease were excluded. In total, four groups of normal valve tissues and four groups of calcified valve tissues were obtained. After valve dissection, we cut the valve in half from the aortic end to the tip, and half of the valve was stored in formalin at −4°C for paraffin embedding, while the other group was placed in liquid nitrogen for RNA and protein extraction. This study was approved by the Research Ethics Committee of Qilu Hospital (reference number: KYLL-2021(KS)-393), in compliance with the Declaration of Helsinki, the Code of Ethics of the World Medical Association. Written consent to participate in the study was obtained from all patients.

### 2.9. Real-time polymerase chain reaction analysis

Relative levels of target genes were measured using real-time polymerase chain reaction (qPCR). Total RNA was extracted from human heart valve tissue using the RNeasy Fibrous Tissue Mini Kit (QIAGEN, Hilden, Germany) according to the manufacturer’s instructions. cDNA was synthesized using the High Capacity cDNA Reverse Transcription Kit (Thermo Fisher Scientific, Waltham, MA). Fluorescence quantification of target genes was performed using the ABI 7500 Fast Real-Time PCR System (Applied Biosystems, Foster City, CA) in a 20 μl reaction mixture, including 1 μL cDNA, 2 μl forward and reverse primers, Fast SYBR Green Master Mix 10 μl (Thermo Fisher Scientific) and 8 μl of nuclease-free water. Primer sequences for qRT-PCR were as follows: SCG2: F, 5′-ACCAGACCTCAGGTTGGAAAA-3′, R 5′-AAGTGGCTTTCAT CGCCATTT-3′; GAPDH: forward, 5′-ATCCCATCACCATCTTCC-3′, and R, 5′-GAGTCCTTCCACGATACCA-3′. ZEB1, F: 5′-ATGG CGGATGGCCCAG-3′, R: 5′-GGCTTCATTTGTCTTTTCTTC-3′.

### 2.10. Western blotting

For protein extraction from human tissues, we used four normal human valves and four calcified valves. The extracted proteins were fractionated by SDS-PAGE (12% polyacrylamide gels). PVDF membranes and primary antibodies with proper concentration were co-incubated overnight at 4 degrees Celsius. The following primary antibodies were used: including anti-SCG2 (1:1000,20357-1-AP, ptglab). PVDF membranes were washed three times (15 min once) in TBST, followed by a reaction with goat anti-rabbit horseradish peroxidase (HRP)-conjugated secondary antibodies (1:6,000) for 2h at room temperature. Finally, protein bands were visualized using enhanced chemiluminescence reagents and analyzed by Graphpad Prism9 software.

### 2.11. Immunohistochemistry staining

For immunohistochemistry (IHC), 5 μm thick sections were deparaffinized and rehydrated using xylene and a graded series of ethanol. Antigen retrieval was performed in 10 mM sodium citrate buffer (pH 6.0), which was microwaved at 100°C for 20 min. Sections were incubated with endogenous peroxide scavenging agents and then blocked using 5% normal rabbit serum, followed by incubation with anti-ZEB1(21544-1-AP, ptglab), anti-SCG2(20357-1-AP, ptglab) overnight at 4°C. The following day, the sections were taken out for reinstatement of temperature and washed with PBST solution, followed by dropwise addition of secondary antibody and incubation for 1 h in an incubator at 37 degrees Celsius. After washing with PBS and adding DAB solution dropwise, brown particles were visible under high-powered microscope.

To calculate the IHC scores, using Image-Pro Plus 6.0, sections of three different individuals were taken at random, and photographs of four randomly selected areas from each slide (magnification, × 200) were measured to obtain the average. All measurements were performed in a single-blind condition. The cumulative optical density (IOD) is the value obtained by summing the optical density values of each brown spot on the image. The mean density is the value obtained by dividing the IOD by the area (region) of the effective target distribution. Immunohistochemical photographs are mainly used to compare the size of the average optical density between groups. The depth and extent of staining patches on an image reflects the degree of expression of a particular protein on the section and can be expressed relatively quantitatively in terms of the integrated optical density on the image. The average optical density, reflects the intensity of expression of the immunoreactant on that photograph. Mean density = (IOD SUM)/area. Mean density and IOD are relative values and therefore unitless.

### 2.12. Statistical analysis

In this study, the GEO dataset was processed and analyzed using R’s online tool site Sangerbox,^7^ and for statistical analysis of PCR and WB data, Prism9 mapping software was used, and ImageJ was used for grayscale statistics, for the statistical analysis of IHC we use Image-Pro Plus 6.0.

## 3. Results

### 3.1. DEGs between the calcified, normal aortic valve and intersected angiogenesis-related genes

**FIGURE 1:**
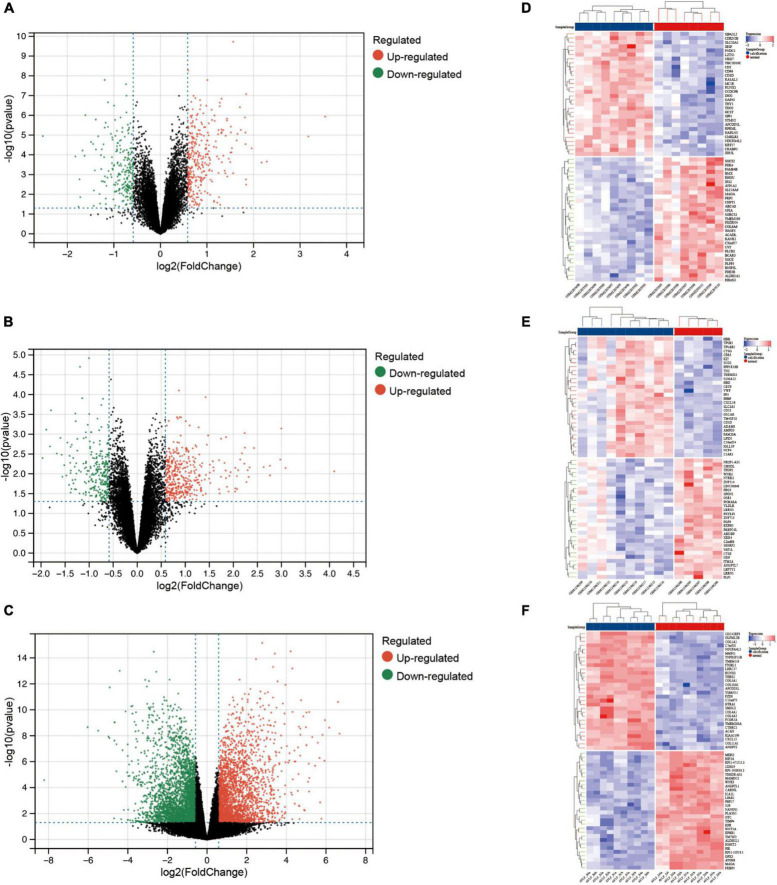
Differentially expressed genes between calcified and normal aortic valve samples. Volcano plots for differential gene expression data from: **(A,D)** GSE83453, **(B,E)** GSE51472, **(C,F)** GSE153555. In the volcano plots and heatmap, the red sections show upregulated genes (log2FC ≥ 0.5 and adjusted *p*-value < 0.05), whereas the green/blue sections represent downregulated genes; black dots represent no significant difference.

**FIGURE 2:**
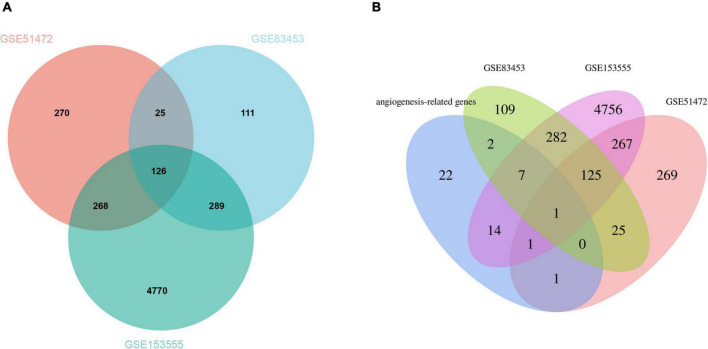
**(A)** The Venn plot of DEGs from GSE83453, GSE51472, and GSE153555. **(B)** Venn plots showing the relationship between DEGs and angiogenesis-related genes across all three gene sets. Venn plots show the relationship between DEGs and angiogenesis-related genes in all three gene sets. Intersected DEGs and angiogenesis-related genes in at least two CAVD datasets were screened as strongly correlated genes, and one key gene was identified.

**FIGURE 3:**
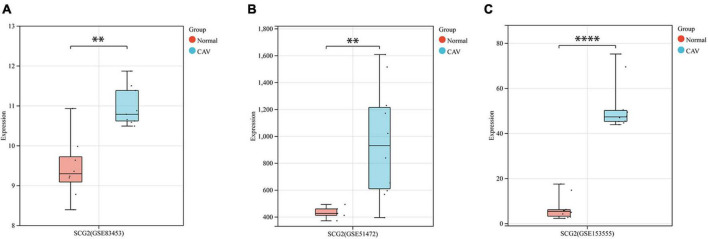
Boxplots represent the gene expression levels of calcified samples compared to normal samples in the three datasets. **(A)** GSE83453, **(B)** GSE51472, **(C)** GSE153555. Wilcoxon rank-sum test, normal, control; CAV, calcified aortic valve. *p < 0.05, ^**^p < 0.01, ^****^p < 0.0001.

After pre-processing datasets GSE83453, GSE51472, and GSE153555, we screened DEGs according to the cutoff criteria (adjusted *P*-value < 0.05 and | log_2_ FC| ≥ 0.5). In GSE83453, we identified 323 upregulated and 228 downregulated genes (Figure 1). In GSE51472, 409 genes were upregulated, and 280 genes were downregulated (Figure 1). A total of 2,323 upregulated and 3,130 downregulated genes were identified in GSE153555 (Figure 1). The volcano plot for all genes and the expression heatmap of the top 30 DEGs are shown in Figure 1. The intersected genes between DEGs and angiogenesis-related genes were selected. A total of nine genes were identified in at least two CAVD datasets and angiogenesis-related genes (Figure 2), including RUNX1, COL4A2, THY1, PF4, SPHK1, COL4A3, ANGPTL4, and RHOB. The SCG2 gene was present in all datasets. Accordingly, SCG2 was considered the most important gene in angiogenesis-related genes that promotes valve calcification. The expression of SCG2 in the three datasets is shown in Figure 3.

### 3.2. Enrichment analysis of DEGs

**FIGURE 4:**
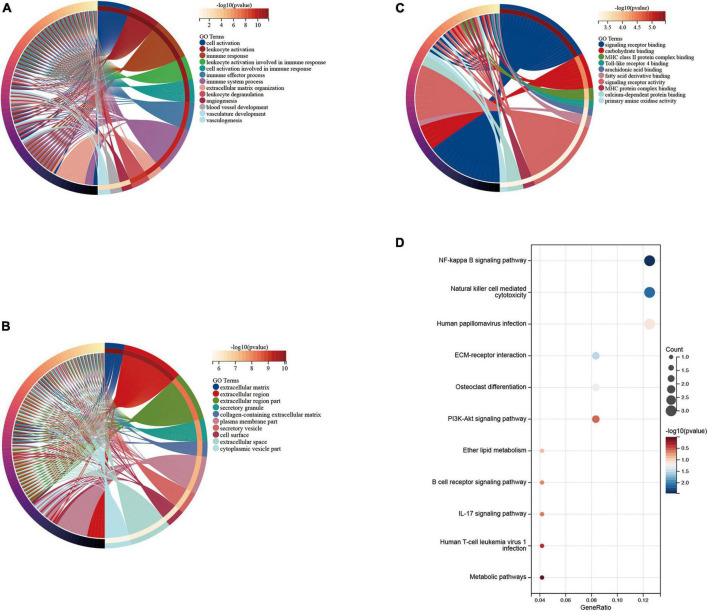
Enrichment analysis of disease genes. Gene Ontology (GO) **(A)** biological process, **(B)** cellular component, **(C)** molecular function, Kyoto Encyclopedia of Genes and Genomes (KEGG) **(D)** enrichment analysis.

We performed functional enrichment analysis of DEGs based on GO and KEGG databases. As shown, gene ontology contains three domains (biological processes, cellular components, and molecular functions). The DEGs were significantly enriched in biological processes, including immune responses, leukocyte activation, immune system processes, and angiogenesis-related pathways (Figure 4A). Significantly enriched cellular components were the extracellular domain, extracellular matrix, part of the extracellular domain and the cell surface (Figure 4B), while enriched molecular functions mainly involved carbohydrate binding, signaling receptor activity, molecular transduction activity and serine-type Endopeptidase activity (Figure 4C). KEGG pathway analysis showed significant enrichment in NF-kappa B signaling pathway, natural killer cell-mediated cytotoxicity, human papillomavirus infection, ECM-receptor interaction, PI3K-Akt signaling pathway and osteoclast differentiation (Figure 4D).

### 3.3. miRNA-target gene regulatory network

**FIGURE 5:**
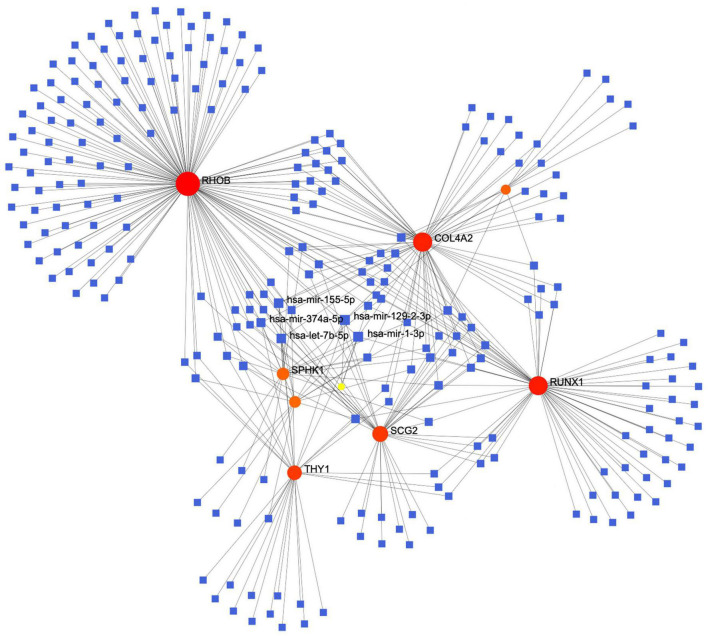
The protein-miRNA interaction network diagram, in which the blue squares represent miRNAs, the orange origins represent the related genes we screened out, with the top five miRNAs with the highest comprehensive scores displayed.

The constructed miRNA-mRNA network consisted of nine key genes. We retrieved data using TarBase v8 miRNA databases and then used NetworkAnalyst to build a co-expression network. The top five highest scoring miRNAs were hsa-mir-155-59, hsa-mir-374a-5p, hsa-mir-1292-3p, hsa-let-7b-5p, and hsa-mir-1-3p (Figure 5).

### 3.4. TF-miRNA coregulatory network

**FIGURE 6:**
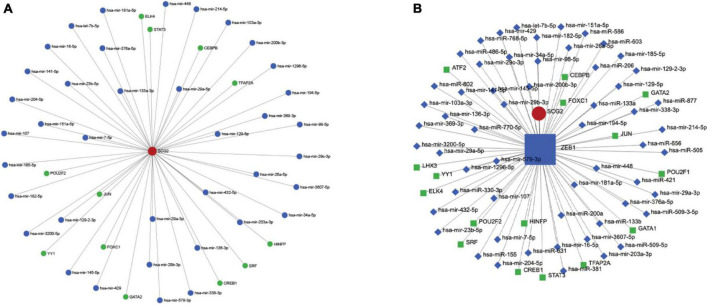
**(A)** Upstream miRNAs and transcription factors of the key gene SCG2. **(B)** Interaction network diagram of the key transcription factor ZEB1.

The hub gene SCG2 underwent gene, TF, and miRNA pathway analysis in TarBase v8.0, JASPAR, and RegNetwork datasets (Figure 6A), respectively. A separate analysis of our hub gene SCG2 was performed. The transcription factor ZEB1 was at a key central location for regulating genes and miRNAs (Figure 6B).

### 3.5. Establishment of the protein-chemical network

**FIGURE 7:**
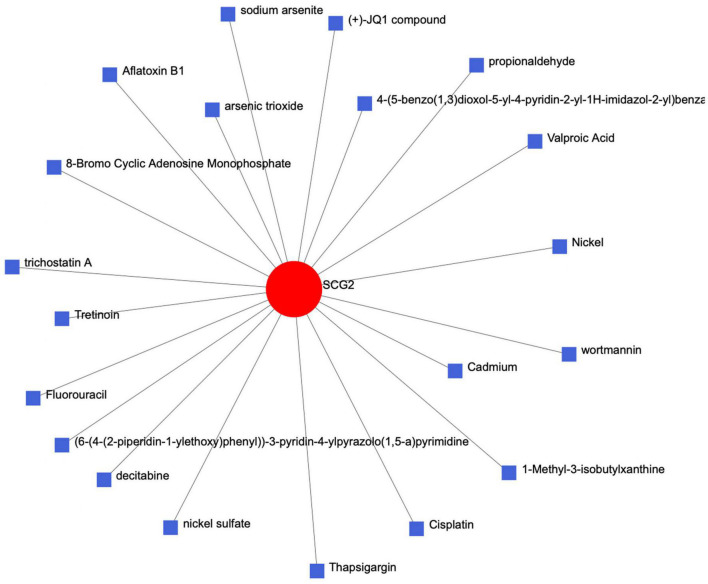
The protein-chemical-molecular interaction network diagram of the key protein gene SCG2.

We entered the key gene SCG2 into the Comparative Toxicogenomics Database (CTD) of the network analyst website to establish a protein chemical interaction network and identified many chemical complexes that could react with SCG2 (Figure 7).

### 3.6. Expression of SCG2 and ZEB1 in human normal and calcified aortic valve

**FIGURE 8:**
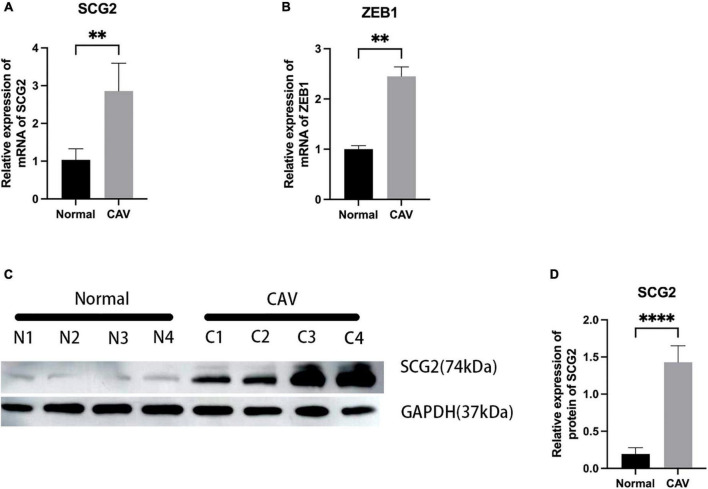
Validation of SCG2 and ZEB1 in the calcified aortic valve. **(A,B)** The mRNA expression levels of SCG2 and ZEB1 in valves from normal patients (*n* = 5 biologically independent samples) and calcification patients (*n* = 5 biologically independent samples) were obtained by RT-PCR and normalized with GAPDH. **(C,D)** The protein expression level of SCG2. The bar graphs represent the expression levels of SCG2 protein in normal and calcified tissues. Valves from normal patients and CAVD patients were validated by Western Blotting. N1-N4 represent different samples of normal individuals, C1-C4 represent four individuals with CAV. ImageJ and GraphPad Prism softwares were used to analyze the expression level of SCG2 protein in normal and calcified aortic valves. Mann–Whitney U, *p < 0.05, ^**^p < 0.01, ^****^p < 0.001, normal, control; CAV, calcified aortic valve.

**FIGURE 9:**
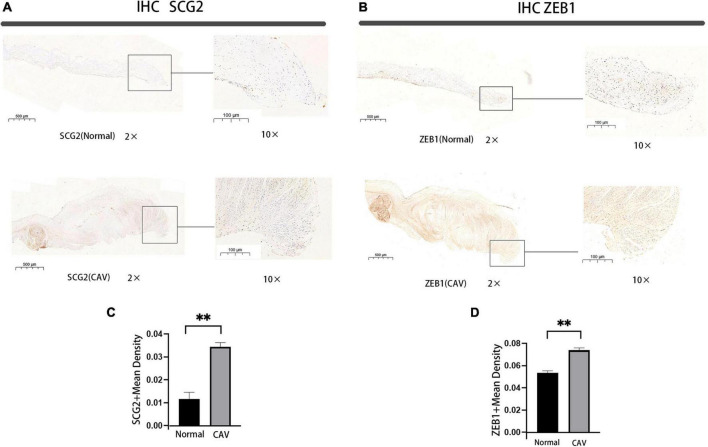
Immunohistochemical staining of SCG2 and ZEB1 in human aortic valve, Bar chart for quantitative assessment of immunostaining. normal: normal aortic valve (*n* = 5 biologically independent samples), CAV: CAVD patients (*n* = 5 biologically independent samples). *p < 0.05, ^**^p < 0.01 **(A,B)** immunohistochemical staining of the panorama of SCG2 and ZEB1. **(C,D)** Comprehensive quantitative expression analysis based on immunohistochemically stained sections.

We performed a qRT-PCR analysis of the mRNA expression of SCG2 and ZEB1 in human normal and calcified aortic valves. As expected, the expression of SCG2 and ZEB1 was significantly increased in the human calcified aortic valve compared with the normal aortic valve. The upregulated expression of SCG2 protein was demonstrated by western blot (Figure 8). After immunohistochemical staining of normal human aortic valve and calcified aortic valve, it was found that the expression of SCG2 and ZEB1 was significantly increased in calcified aortic valve compared with normal human aortic valve (Figure 9).

## 4. Discussion

Although there have been numerous investigations on CAVD, the precise mechanisms are still unknown. Indeed, understanding molecular pathways is essential for early diagnosis and treatment. In the present study, GO analysis of DEGs showed significant enrichment in immune response, leukocyte activation, immune system process, and extracellular matrix organization. In addition, KEGG analysis showed that the DEGs were mainly enriched in the NF-kappa B signaling pathway, PI3K-Akt signaling pathway, ECM-receptor interaction, human papillomavirus infection, and osteoclast differentiation. It has been established that angiogenesis-related factors play an important role in various diseases such as coronary atherosclerosis (18), tumor growth and metastasis (19). The three datasets were subjected to differential expression analysis, and the intersection of the three datasets produced eight linked genes, including RUNX1, COL4A2, THY1, PF4, SPHK1, COL4A3, ANGPTL4, and RHOB. Furthermore, the significant gene SCG2 was discovered.

Neovascularization, ECM abnormalities, inflammation, angiogenesis and fibrotic thickening of the valve leaflets are characteristics of CAVD (3, 7). Current research implies that numerous pathophysiological alterations precede calcification of the aortic valve (20). In this regard, a range of inflammatory cells including macrophagocytes, leukocytes, dendritic cells, mastocytes and thrombocytes were discovered in CAVD tissues (21). In addition, a research indicated that around 95% of calcified aortic valves had significant inflammatory infiltration (22). Leukocyte density was found to be associated to the advancement of aortic valve stenosis, according to Cote et al. (23). The interaction between angiogenesis and inflammation may play a significant role in the development of CAVD (24). Angiogenesis-related factors are highly likely to be important factors in the progression of CAVD. Recent research has suggested that an imbalance between pro- and anti-angiogenic factors contributes to the evolution of CAVD pathological processes (12). Numerous studies have already shown that angiogenesis-related variables play a crucial role in the pathogenesis of CAVD (25, 26). Interestingly, these conclusions share many similarities with our findings, given that GO analysis in the present study showed that DEGs in the three datasets were enriched in immune-inflammatory pathways and angiogenesis-related pathways.

Kyoto Encyclopedia of Genes and Genome analysis revealed that the PI3K-AKT and NF-kappa B signaling pathways were considerably enriched. Numerous studies indicate that the PI3K-AKT and NF-kappa B signaling pathways are essential for the development of numerous illnesses (24, 25). Other research groups findings accord with ours in highlighting the significance of the aforementioned pathways in CAVD (26–28). The specific mechanism underpinning the aforementioned CAVD pathways is yet not clearly realized. According to studies on the problem, a number of medications can stop the activation of the aforementioned two pathways and slow down the calcification of the interstitial cells in the aortic valve (27). It is uncertain how the PI3K-AKT and NF-kappa B signaling pathways contribute to the evolution of CAVD, although a number of *in vitro* studies have demonstrated that variations in NF-kappa B pathway (25–27) and PI3K-AKT pathway (28, 29) activity play an important role in CAVD. Secretogranin II (SCG2) is a 587-amino acid member of the chromogranin-secretogranin protein family (28). Through the intersection of DEGs and angiogenesis-related genes in the three datasets, we identified SCG2 as a potential angiogenic factor for CAVD. SCG2 has been shown to serve as a pro-angiogenic agent and boost vascular endothelial growth factor (VEGF) signaling in endothelial cells, resulting in postnatal angiogenesis (26), as well as promote the buildup of inflammatory cells/macrophages and stimulate vascular smooth muscle cell (VSMC) proliferation (27). It may contribute to the development of CAVD by angiogenesis and inflammation.

Indeed, miRNAs can inhibit target genes from expressing themselves post-transcriptionally, which is crucial for the beginning and progression of a variety of diseases. Significantly, suppression of miR-155-5p has been demonstrated to minimize the valve damage caused by rheumatic heart disease (29). MiR-155p is important for systemic sclerosis and positively correlates with the severity of atherosclerotic plaque in coronary atherosclerosis, according to research (30, 31). Inflammatory factors like TNF, IL1A, IL6, and others are regulated by miRNAs, and a study on this topic found that mir-374a-5p is essential for this process (32). Additionally, let-7b-5p is essential for plaque inflammation in coronary atherosclerosis (30). Mir-129-2-3p is related with the recruitment of inflammatory cells such as neutrophils in wounds that heal slowly (31). MiR-1-3p regulates the Notch3-Smad axis to regulate several inflammatory diseases in humans (32). It’s interesting to note that the NOTCH gene axis has a crucial role in the mutation of calcification-prone congenital bicuspid valve defects (33). It should be remembered that dysregulation of the ECM plays an important part in the development of CAVD. Recent research has shown that non-coding RNAs play a crucial part in the regulation of the ECM. In disorders linked to ECM disruption or breakdown, several miRNAs, including miR-140, miR-29, miR-30, and miR-133, have been severely dysregulated (33). Many studies have demonstrated that the pathogenic mechanisms and pathways generating calcification of the aortic valve and coronary atherosclerosis are comparable (11). Although many miRNAs have not been identified in CAVD-related pathways, it has been demonstrated that they play a crucial role in certain fibrotic disorders. The influence of these miRNAs has been investigated in prior research. This study’s identification of various miRNAs may yield novel insights into the pathophysiology of CAVD.

Trans-acting components known as transcription factors (TFs) are involved actively in the transcription process. Transcriptional start is aided by transcription factors, which RNA polymerase needs (34). Here, we investigated the interaction networks between TFs and miRNAs associated with the screening essential gene SCG2. We found that Zinc finger E-box binding homeobox 1 (ZEB1), a key transcription factor of SCG2, is in the most critical position. ZEB1 is an essential transcription factor in epithelial-mesenchymal transition (EMT), which is involved in embryonic development, fibrosis, and tumor progression, among other biological processes (35). Osteoblastic trans-differentiation of aortic valve interstitial cells (VICs) is a crucial step in the development of CAVD (36, 37). Aortic VICs has been demonstrated to result from the EMT (38). ZEB1 can stimulate cell growth, migration, and collagen production while inducing fibrosis *via* EMT (39–41). By stimulating the PI3K-AKT signaling cascade and activator of transcription in lung tissue, ZEB1 has been demonstrated to increase the progression of pulmonary fibrosis (42, 43). Besides, ZEB1 reportedly upregulate in liver fibrosis (44). The studies mentioned above and our findings suggest that ZEB1 may play an important role in fibrosis in human tissues.

Moreover, in the present study, we investigated the effect of angiogenesis-related factors on aortic valve calcification and discovered that SCG2 and ZEB1 may be the key aortic valve calcification factors. SCG2 and ZEB1 were significantly upregulated in the calcified aortic valve (CAV) relative to the normal group. SCG2 may be one of the major factors that promote calcification of the aortic valve. Given that ZEB1 is a key transcription factor of SCG2, we hypothesize that the upregulation of ZEB1 may result in the upregulation of SCG2, thereby indirectly influencing the pathophysiological process of CAVD. In addition, the preceding studies demonstrated that numerous non-coding RNAs may play an important role in regulating calcification of the aortic valve. SCG2 and ZEB1 are significant influencing factors for aortic valve calcification, providing novel ideas for the diagnosis and treatment of CAVD patients.

Several limitations were present in our study. For instance, we did not use strict inclusion criteria for differentially expressed genes and we relaxed the criteria for the analysis of individual valves with calcification. In the initial data analysis, we used some sclerotic valves that were less calcified than the severely calcified valves, and the inclusion of these samples may have led to some errors in the data analysis, for example, some genetic alterations in the severely calcified valves may have been overlooked, but by including these sclerotic valves, we may also have found genetic alterations associated with minor calcification. Moreover, we did not conduct cell culture and animal experiments to validate the impact of specific signaling pathways. Based on our findings, we hypothesize that in non-vascular tissues, factors related to angiogenesis have an important impact on the progression of the disease. Besides, we validated our findings in CAV and normal human aortic valve. In conclusion, we identified two genes that play a significant role in aortic valve calcification; however, the underlying pathological pathways remain unclear and require additional study.

## Data availability statement

The datasets presented in this study can be found in online repositories. The names of the repository/repositories and accession number(s) can be found in this article/supplementary material.

## Ethics statement

The studies involving human participants were reviewed and approved by the Qilu Hospital (reference number: KYLL-2021(KS)-393). The patients/participants provided their written informed consent to participate in this study.

## Author contributions

XK performed the data collection and analysis. XK and LM were involved in the validation of the basic experiment. CL critically revised the manuscript. XG provided the financial support. All authors contributed to the design of the ideas, conceived and designed the study, contributed to the article, and approved the submitted version.

## Footnotes

1
https://www.gsea-msigdb.org/gsea

2
https://www.tarbase.com

3
https://jaspar.genereg.net

4
https://www.encodeproject.org

5
https://www.networkanalyst.ca/home.xhtml

6
https://ctdbase.com

7
http://sangerbox.com/login.html
